# Electrical Alternans as an Early Clue to a Rapidly Evolving Large Pericardial Effusion: The Role of Point-of-Care Ultrasound

**DOI:** 10.7759/cureus.117215

**Published:** 2026-09-30

**Authors:** Mohamad Zbidat, Mamoun Bashir

**Affiliations:** 1 Emergency Department, Western Galilee Hospital, Nahariya, ISR

**Keywords:** acute pericarditis, cardiac tamponade, electrical alternans, pericardial effusion, point-of-care ultrasound

## Abstract

Electrical alternans is an uncommon electrocardiographic finding classically associated with significant pericardial effusion and may raise suspicion for cardiac tamponade in the appropriate clinical context.

We report the case of a 65-year-old man who presented with 3 days of progressive dyspnea 10 days after hospitalization for acute pericarditis, during which transthoracic echocardiography showed no pericardial effusion. On presentation, he was tachycardic at 110 beats/min and had jugular venous distension, while electrocardiography demonstrated prominent electrical alternans. Although he was not hypotensive and had no overt evidence of hemodynamic compromise, the combination of recent pericarditis, progressive dyspnea, venous congestion, and electrical alternans raised immediate concern for a rapidly evolving large pericardial effusion with clinical features suggestive of cardiac tamponade. Point-of-care ultrasound rapidly demonstrated a large pericardial effusion, prompting urgent transfer for definitive evaluation and management. Within minutes of transfer, the patient's respiratory distress worsened despite persistently preserved vital signs, and urgent pericardial drainage was performed. Approximately 1,200 mL of grossly hemorrhagic pericardial fluid was evacuated, resulting in rapid and marked clinical improvement.

This case highlights electrical alternans as an important early clinical clue to significant pericardial effusion and emphasizes the value of immediate point-of-care ultrasound in the emergency department when cardiac tamponade is clinically suspected. It also demonstrates the dynamic nature of pericardial disease, showing that a large pericardial effusion with clinical features suggestive of cardiac tamponade may develop within a short interval after a previously negative echocardiographic examination. Integrating electrocardiographic findings with rapid bedside ultrasound assessment may facilitate earlier recognition of evolving clinically significant pericardial disease and timely definitive intervention.

## Introduction

Cardiac tamponade is a potentially life-threatening clinical syndrome resulting from the accumulation of pericardial fluid, blood, pus, or air within the pericardial space, leading to impaired cardiac filling and, in severe cases, hemodynamic compromise. The clinical presentation depends not only on the absolute volume of pericardial fluid but also on the rate at which it accumulates and the ability of the pericardium to accommodate the increasing pressure. Consequently, patients with significant pericardial effusion may initially present without the complete classical clinical features of cardiac tamponade, particularly when the process evolves rapidly or before overt hemodynamic compromise develops. Echocardiography remains the principal imaging modality for evaluating pericardial effusion and its hemodynamic consequences [1].

Electrical alternans is an uncommon electrocardiographic finding characterized by beat-to-beat variation in QRS amplitude. It is classically associated with large pericardial effusions and cardiac tamponade, although it is not specific to tamponade and may occur in other cardiac conditions. One proposed mechanism is the mechanical “swinging heart” phenomenon, in which the heart moves within a large pericardial effusion, producing cyclic changes in its position relative to the surface ECG electrodes and consequent variation in QRS amplitude [2]. Importantly, electrical alternans should therefore be interpreted in the context of the patient’s clinical presentation and other diagnostic findings rather than as an isolated finding diagnostic of cardiac tamponade.

Point-of-care ultrasound (POCUS) provides a rapid bedside method for identifying pericardial fluid and can be particularly valuable in emergency settings when a patient develops new or worsening symptoms. The integration of POCUS with the clinical examination and ECG may facilitate earlier recognition of a clinically significant pericardial effusion and help expedite definitive evaluation and management.

The present case highlights the potential for a large pericardial effusion to develop over a relatively short period despite a recent echocardiographic examination showing no pericardial effusion and despite initiation of appropriate treatment for acute pericarditis. It further illustrates the importance of integrating evolving clinical findings and ECG abnormalities, including prominent electrical alternans, with early bedside POCUS rather than relying on any single finding in isolation. In this case, POCUS facilitated rapid identification of the large pericardial effusion in the setting of clinical deterioration, allowing timely definitive intervention. The case also underscores the diagnostic limitations of establishing cardiac tamponade solely based on pre-drainage POCUS when the specific quantitative echocardiographic parameters required for definitive echocardiographic assessment are unavailable.

## Case presentation

A 65-year-old man presented to our emergency department with a 3-day history of progressively worsening dyspnea. His medical history was significant for type 2 diabetes mellitus and psoriasis, for which he had been receiving ixekizumab (Taltz®) injections once monthly for several years. His diabetes was treated with oral sitagliptin/metformin (Janumet® 50/850 mg), taken once or twice daily.

Ten days before his presentation to our institution, the patient had been hospitalized at another medical center because of pleuritic chest pain and dyspnea. At that time, physical examination revealed no pericardial friction rub or clinical signs of heart failure. The electrocardiogram (ECG) demonstrated sinus rhythm with diffuse PR-segment depression and widespread ST-segment elevations, consistent with acute pericarditis. Laboratory investigations showed mild leukocytosis (11,000/μL) and an elevated C-reactive protein (CRP) level of 15 mg/dL (reference range, 0-5 mg/dL), while serial troponin measurements were negative.

A POCUS examination performed in the emergency department demonstrated a small pericardial effusion, which was considered physiologic, with no echocardiographic features suggestive of cardiac tamponade. On the second day of hospitalization, a formal transthoracic echocardiogram (TTE) demonstrated preserved left and right ventricular systolic function, minimal mitral valve thickening, and no evidence of pericardial effusion.

The patient was discharged on the second hospital day with a diagnosis of acute pericarditis and was prescribed ibuprofen 600 mg 3 times daily for 4 weeks, with a planned gradual dose reduction after the second week, and colchicine 0.5 mg twice daily for at least 3 months, with reassessment thereafter.

Following discharge, the patient initially remained at home; however, approximately 10 days later, he developed progressively worsening dyspnea. His symptoms had intensified over the preceding three days before presentation to our emergency department, prompting him to seek further medical evaluation.

On arrival at our emergency department, his vital signs were relatively preserved. His blood pressure was 124/64 mmHg, heart rate approximately 110 beats/min, respiratory rate 18 breaths/min, and oxygen saturation 95% on room air. He was afebrile (36.6 °C). Despite the absence of hypotension or significant oxygen desaturation, the patient appeared clinically symptomatic, with increased work of breathing, subtle use of accessory respiratory muscles, and mild difficulty completing full sentences because of dyspnea.

Physical examination revealed jugular venous distension and distant, muffled heart sounds. Pulmonary auscultation revealed clear bilateral breath sounds without wheezing or crackles and absence of peripheral edema.

An ECG obtained on arrival demonstrated sinus tachycardia at approximately 110 beats/min with a normal cardiac axis and no significant ST-segment or T-wave abnormalities suggestive of acute myocardial ischemia. Notably, beat-to-beat variation in QRS amplitude was observed, consistent with electrical alternans. The QRS complexes were of low voltage, measuring <10 mm in the precordial leads and <5 mm in the limb leads. The PR interval was preserved, without significant prolongation, and the QT interval was not prolonged (Figure 1).

**Figure 1:**
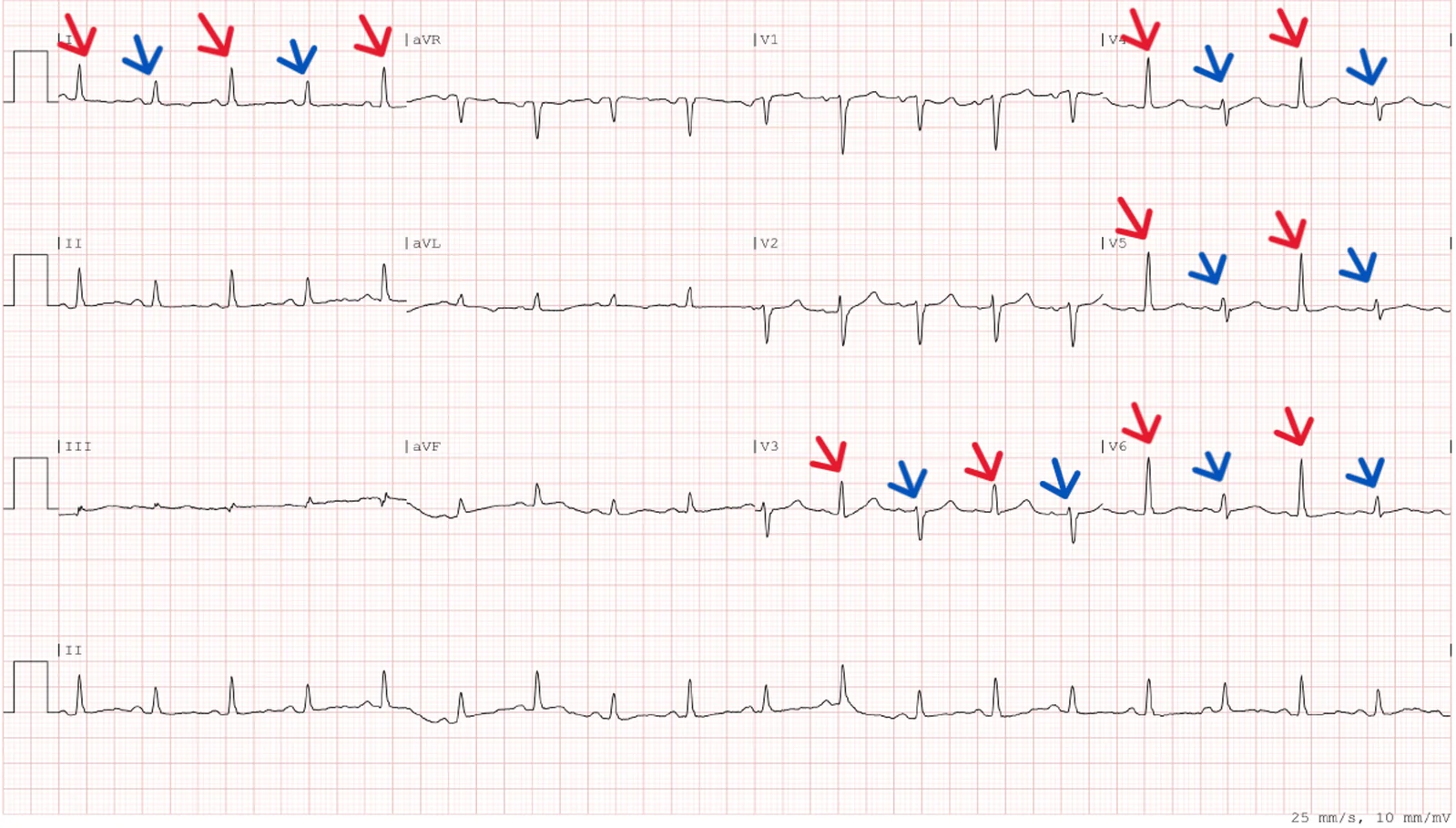
Electrocardiogram demonstrating sinus rhythm with prominent beat-to-beat variation in QRS complex amplitude (electrical alternans), with alternating complexes indicated by red and blue arrows

A chest radiograph obtained on arrival to the emergency department and prior to pericardial drainage was performed in the anteroposterior (AP) projection (Figure 2). Although assessment of cardiac size on an AP radiograph is limited and does not reliably establish cardiomegaly, the cardiac silhouette appeared markedly enlarged even considering the AP technique, which was concerning in a patient without a known cardiac history. The right costophrenic angle appeared mildly obscured, likely related to the technical conditions of image acquisition. Despite the limitations of the AP projection, the marked enlargement of the cardiac silhouette was concerning for significant pericardial effusion.

**Figure 2:**
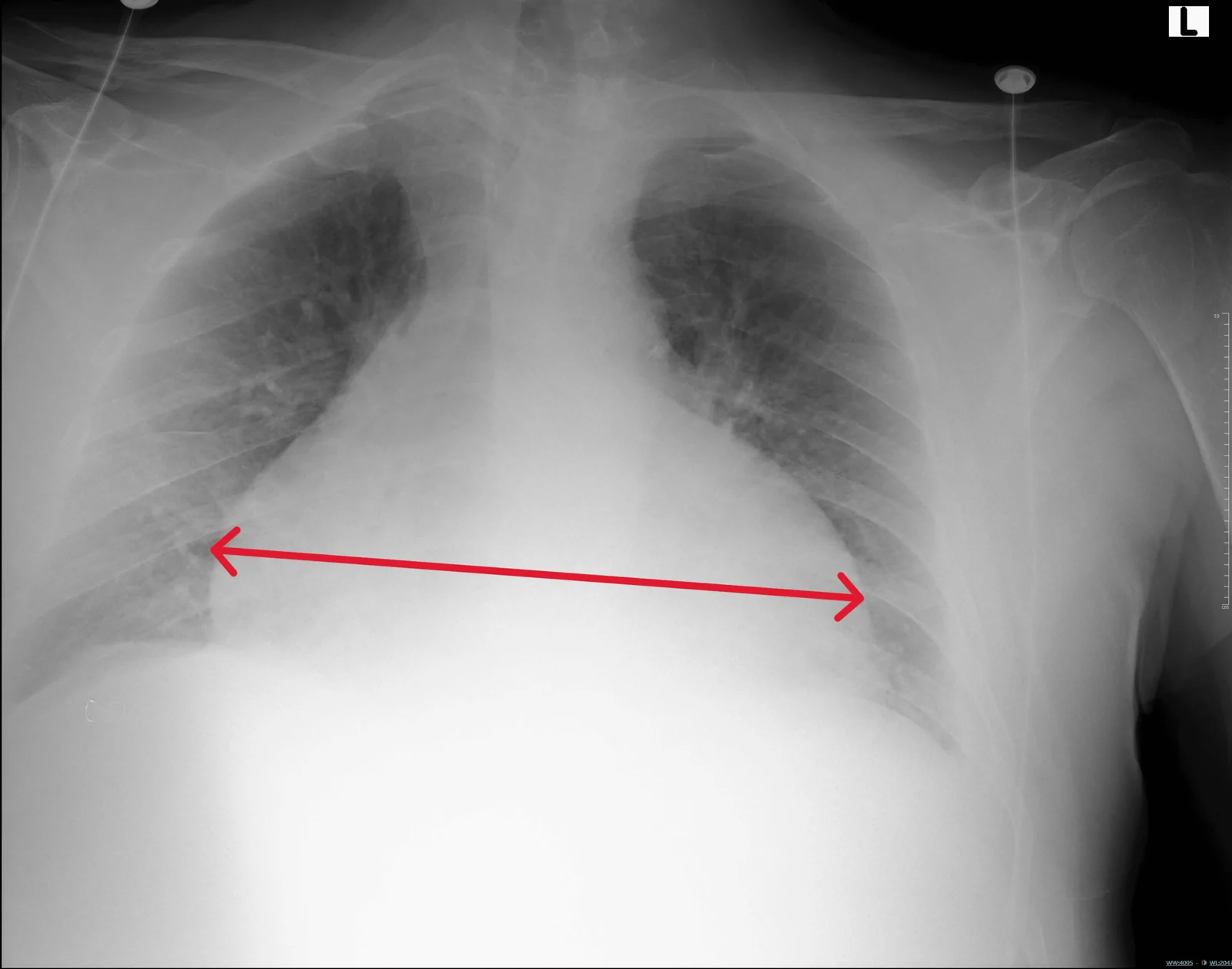
Chest radiograph obtained on arrival to the emergency department prior to pericardial drainage, performed in the anteroposterior (AP) projection, demonstrating a markedly enlarged cardiac silhouette (red double-headed arrow)

Considering the patient's recent history of acute pericarditis, progressive dyspnea over the preceding three days, jugular venous distension, distant heart sounds, and the newly observed ECG abnormalities, the possibility of a large pericardial effusion with clinical features suggestive of cardiac tamponade was raised [2,3]. Given the potential for rapid clinical deterioration and the discrepancy between the patient's relatively preserved hemodynamic parameters and his clinical findings, bedside point-of-care ultrasound (POCUS) was performed almost immediately after arrival.

A subxiphoid cardiac POCUS view demonstrated a large circumferential pericardial effusion, appearing as an anechoic space surrounding the heart, with prominent cardiac swinging motion within the pericardial fluid (Figures 3, 4). This sonographic finding, together with the low QRS voltage and beat-to-beat variation in QRS amplitude observed on the ECG, supported the presence of a large pericardial effusion with clinical features suggestive of evolving cardiac tamponade.

**Figure 3:**
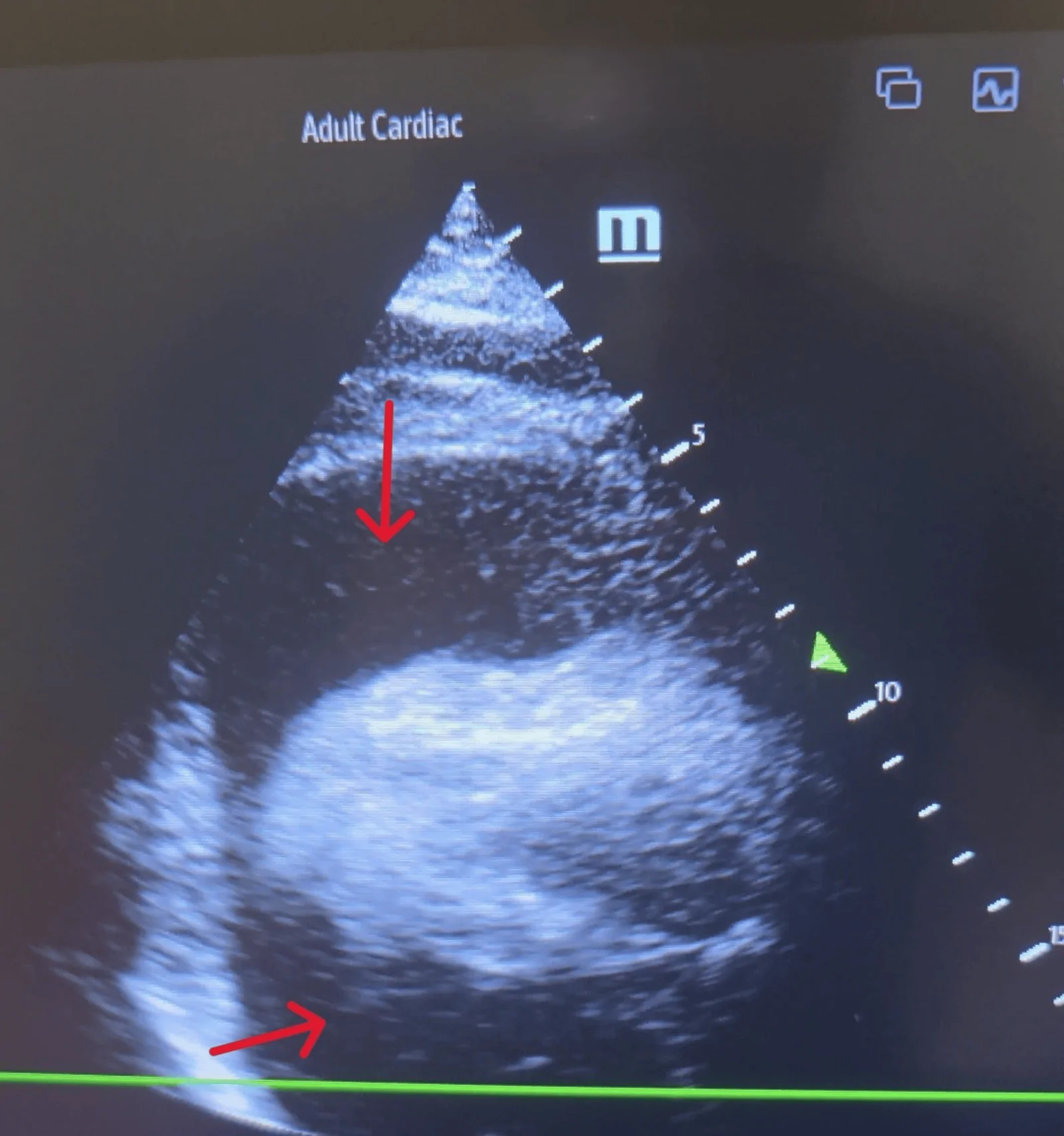
Point-of-care ultrasound demonstrating a large pericardial effusion, visualized as an anechoic (black) fluid collection surrounding the heart (red arrows)

**Figure 4:**
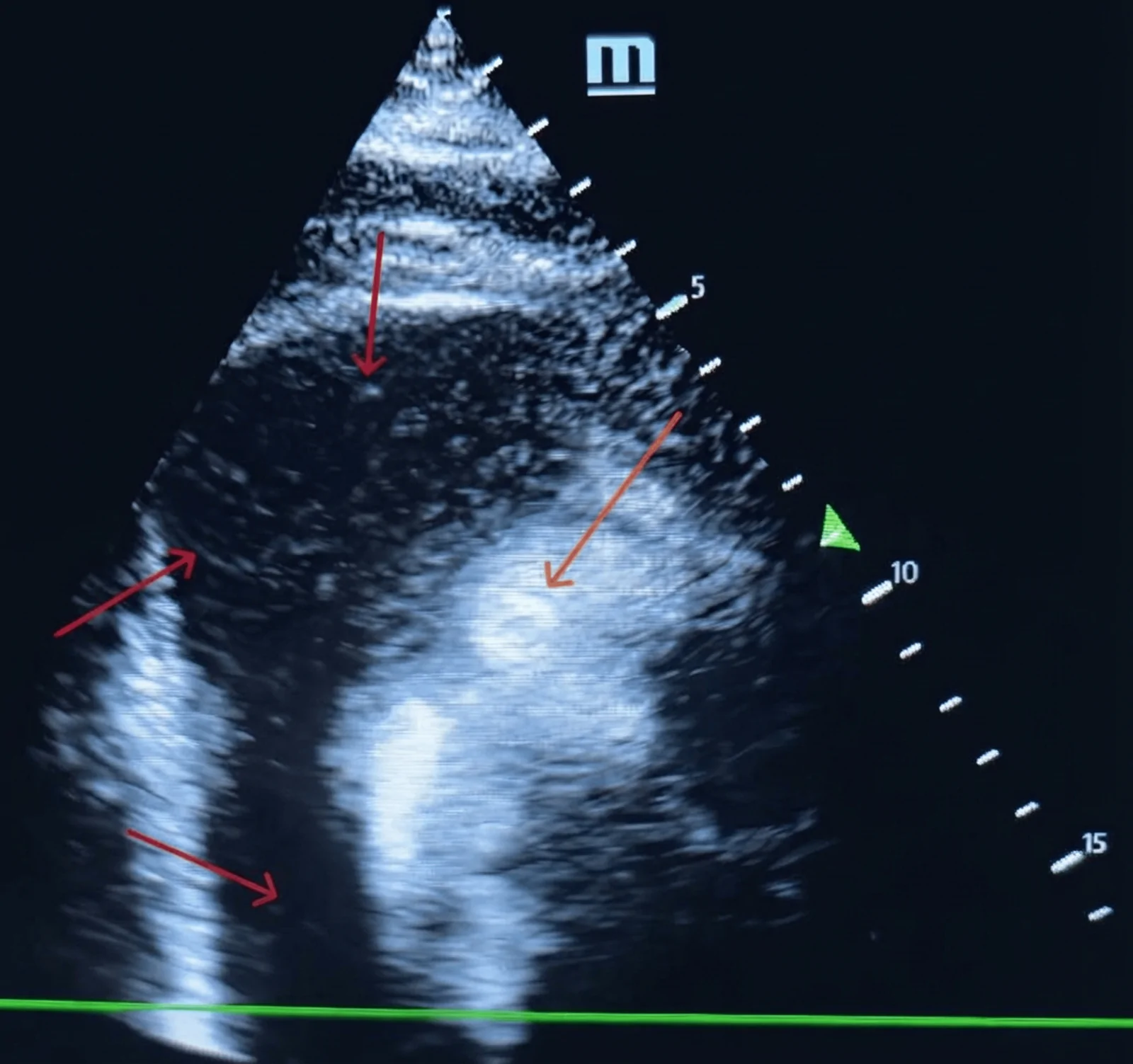
Subxiphoid POCUS view demonstrating a large pericardial effusion (yellow arrow) surrounding the heart (orange arrow) POCUS: point-of-care ultrasound

In the context of the patient's progressive dyspnea, jugular venous distension, distant heart sounds, and recent episode of acute pericarditis, the POCUS findings raised strong concern for a large pericardial effusion with clinical features suggestive of cardiac tamponade.

Given the large pericardial effusion identified on POCUS, together with clinical features suggestive of cardiac tamponade, the patient was transferred immediately to the cardiac intensive care unit for further evaluation and management. Within minutes of arrival, his respiratory distress worsened, with increased work of breathing, marked use of accessory respiratory muscles, and progressive dyspnea.

Notably, despite this clinical deterioration, his vital signs remained within the normal range, without hypotension, significant oxygen desaturation, or other clinical evidence of hemodynamic compromise. In view of this clinical deterioration, urgent pericardial drainage was performed under cardiac ultrasound guidance using a dedicated pericardiocentesis needle. Approximately 1,200 mL of grossly hemorrhagic pericardial fluid was evacuated. There was no history of trauma or clinical suspicion of vascular injury or rupture to explain the hemorrhagic nature of the effusion.

Immediately following drainage, the patient demonstrated marked clinical improvement, with a reduction in respiratory rate and substantially decreased use of accessory respiratory muscles. The pericardial fluid was submitted for microbiological cultures, cytological and histopathological examination, and biochemical analysis, including lactate dehydrogenase (LDH) and protein levels. Microbiological cultures were negative, while cytological and histopathological examination revealed no malignant cells or other findings suggestive of a specific underlying etiology. Pericardial fluid LDH and protein levels were within normal limits. The etiological evaluation was further expanded during hospitalization with computed tomography (CT) of the chest and abdomen, which revealed no evidence of an underlying infectious, inflammatory, malignant, or other secondary process. Based on the overall clinical course and the absence of an identifiable secondary cause, the final diagnosis was considered idiopathic pericarditis complicated by a large pericardial effusion with clinical features suggestive of cardiac tamponade.

## Discussion

Electrical alternans is an uncommon electrocardiographic finding characterized by beat-to-beat variation in QRS complex amplitude. It is classically associated with large pericardial effusions and may occur in the setting of cardiac tamponade [2,3]. The proposed mechanism involves pendular or swinging motion of the heart within the pericardial fluid, resulting in alternating electrical vectors on the surface ECG [2]. Electrical alternans may involve the QRS complexes alone or, less commonly, the P waves and T waves as well. In the appropriate clinical setting, recognition of electrical alternans should raise clinical suspicion for significant pericardial effusion and possible cardiac tamponade and prompt urgent cardiac imaging.

In the present case, a 65-year-old man presented to the emergency department with 3 days of progressive dyspnea 10 days after hospitalization for acute pericarditis, during which transthoracic echocardiography showed no pericardial effusion. His vital signs were within normal limits except for tachycardia at 110 beats/min, and jugular venous distension was noted. The presence of prominent electrical alternans in the setting of recent pericarditis and progressive dyspnea prompted immediate evaluation for pericardial effusion and possible cardiac tamponade. Importantly, clinically significant pericardial effusion with features suggestive of tamponade may develop with a subacute presentation, and the absence of hypotension or other classic clinical findings does not necessarily exclude clinically important pericardial disease [4-8]. Therefore, electrical alternans in a patient with compatible symptoms should be regarded as an important clinical warning sign that warrants prompt cardiac imaging rather than being interpreted in isolation.

The temporal evolution in the present case is particularly noteworthy. Although transthoracic echocardiography performed during the patient's recent hospitalization for acute pericarditis demonstrated no pericardial effusion, a large effusion had developed only 10 days later, accompanied by clinical features suggestive of cardiac tamponade. This highlights the dynamic nature of pericardial disease and demonstrates that a previously negative echocardiographic examination does not exclude subsequent development of a clinically significant effusion. Delayed or subacute cardiac tamponade has been described in several clinical settings, with symptoms sometimes developing gradually rather than presenting with immediate hemodynamic collapse [9-11]. In a previously reported case, a patient developed delayed tamponade after an initially reassuring evaluation and subsequently presented with dyspnea, electrical alternans, and an enlarged cardiac silhouette, with echocardiography demonstrating a new large pericardial effusion [9]. Although the underlying etiology in that case differed from the present case, the temporal pattern illustrates an important clinical principle: pericardial effusion and features suggestive of tamponade may evolve after an initially negative imaging study. In patients with recent pericarditis who develop new or progressive dyspnea, chest discomfort, tachycardia, or venous congestion, repeat cardiac imaging should therefore be considered when clinically indicated.

POCUS performed in the emergency department rapidly demonstrated a large pericardial effusion, facilitating prompt escalation of care and urgent definitive management. POCUS provides a rapid, focused bedside assessment that can be particularly valuable in the emergency department when cardiac tamponade is suspected [4,12-15]. Sonographic findings that may support tamponade physiology include pericardial effusion accompanied by right atrial systolic collapse, right ventricular diastolic collapse, a dilated inferior vena cava with reduced respiratory variation, and significant respiratory variation in transvalvular flow [10,12-14]. Importantly, the size of a pericardial effusion alone does not necessarily determine the presence or severity of tamponade physiology, and sonographic findings should be interpreted together with the clinical presentation [10,13]. In the present case, the initial POCUS examination was performed as a rapid bedside assessment and demonstrated a large pericardial effusion. A complete formal assessment of tamponade physiology, including quantitative evaluation of inferior vena cava dynamics or Doppler respiratory variation, was not documented. Accordingly, the ultrasound findings in this case should be interpreted as demonstrating a large pericardial effusion in the context of clinical features suggestive of cardiac tamponade, rather than as independently establishing tamponade physiology.

In this setting, recognition of electrical alternans may serve as an important clinical trigger for early POCUS, allowing the ECG finding to directly guide further bedside evaluation. This complementary approach may shorten the diagnostic pathway and facilitate timely intervention [12,15]. Recent case reports and case series have highlighted the value of early cardiac POCUS in patients with suspected tamponade, particularly in emergency and acute-care settings where rapid bedside imaging can facilitate earlier recognition of pericardial effusion and potential hemodynamic compromise [12-15]. The role of POCUS in this context is not to replace comprehensive echocardiography but to rapidly identify a clinically significant effusion and, when assessed, features that may support tamponade physiology, thereby supporting early escalation of care.

Following identification of the large effusion, approximately 1,200 mL of pericardial fluid was urgently evacuated with the volume measured using a dedicated graduated blood collection bag connected directly to the pericardiocentesis needle, resulting in rapid and marked clinical improvement. The immediate improvement in respiratory distress following drainage supports the clinical significance of the effusion and the decision to proceed with urgent drainage. The combination of a newly developed large pericardial effusion, clinical features suggestive of impaired venous return, prominent electrical alternans, and rapid clinical improvement following evacuation illustrates the importance of integrating multiple clinical and diagnostic findings rather than relying on a single parameter.

Cardiac POCUS can be performed rapidly using a limited set of standard echocardiographic views and can provide immediate information regarding pericardial effusion, cardiac anatomy, and, when systematically assessed, hemodynamic status, making it particularly valuable in patients presenting with dyspnea or other features suggestive of acute cardiovascular compromise [16]. Moreover, POCUS may facilitate recognition of clinically significant pericardial effusion and features suggestive of cardiac tamponade even when classic clinical findings are incomplete or absent, and its portability and rapid image acquisition can support earlier escalation of care and definitive intervention when clinically indicated [17]. In this context, electrical alternans may serve not only as an electrocardiographic finding associated with significant pericardial effusion, but also as a practical trigger for immediate bedside ultrasound assessment. This integrated ECG-POCUS approach may facilitate earlier recognition of evolving clinically significant pericardial disease and timely definitive management [16,17].

Following pericardial drainage, a complete transthoracic echocardiographic examination demonstrated preserved cardiac contractility with no residual pericardial effusion. A post-drainage electrocardiogram (Figure 5) showed sinus rhythm at approximately 100 beats/min without evidence of persistent electrical alternans.

**Figure 5:**
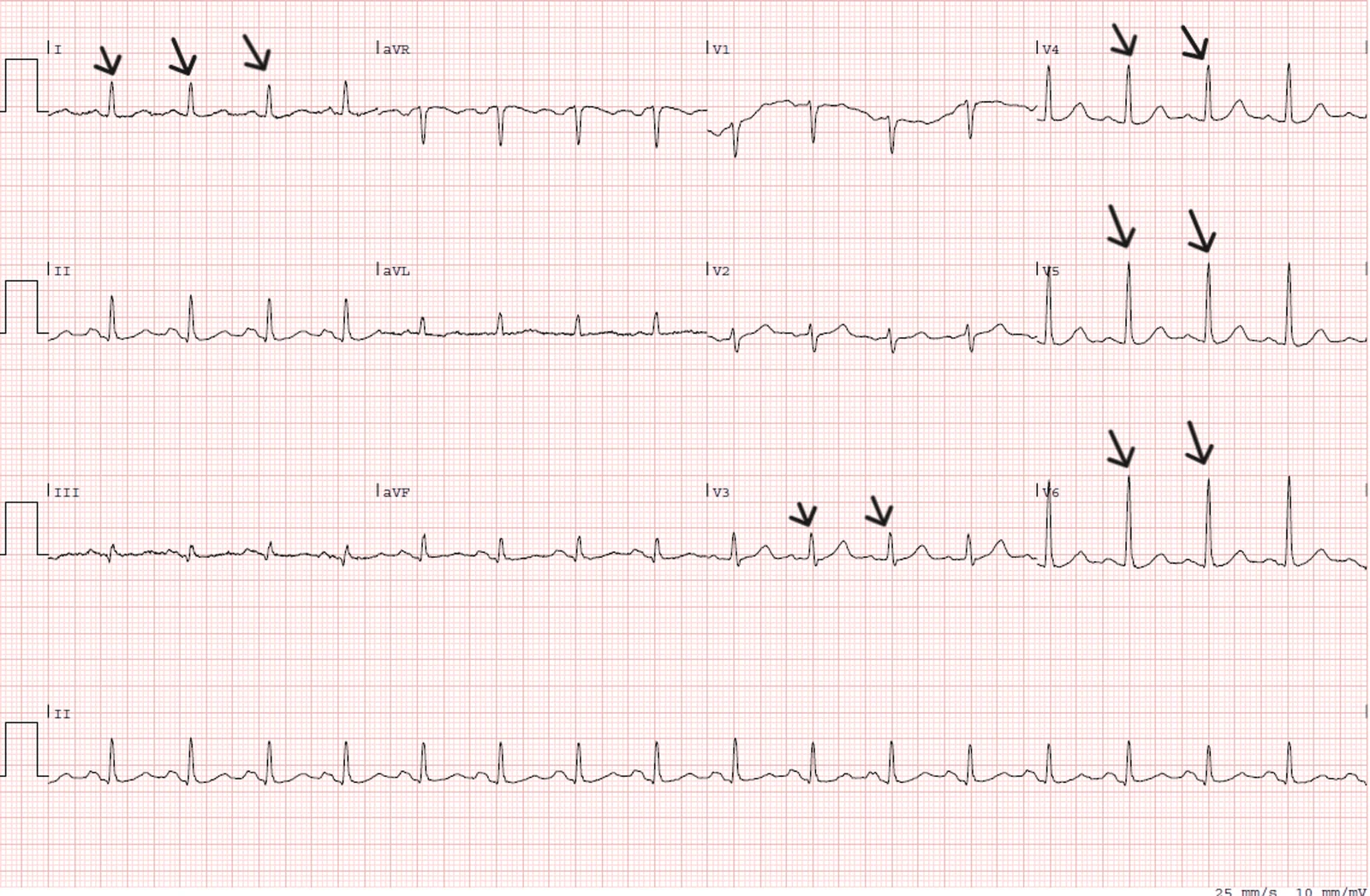
The post-drainage ECG showed regular sinus rhythm at approximately 100 beats per minute, with a normal axis, normal QRS complexes, and complete resolution of electrical alternans (black arrows)

These findings provide objective post-intervention documentation of the resolution of the large pericardial effusion and the associated electrocardiographic abnormality. The patient was discharged with a plan for repeat clinical assessment and follow-up transthoracic echocardiography in the outpatient setting one month after discharge, allowing for reassessment of cardiac function and surveillance for recurrence of pericardial effusion.

## Conclusions

This case highlights the importance of maintaining a high index of suspicion for clinically significant pericardial disease when electrical alternans is accompanied by progressive dyspnea, jugular venous distension, and other compatible clinical findings, even in the absence of hypotension or overt hemodynamic compromise. In this setting, these findings may indicate the presence of a large pericardial effusion with clinical features suggestive of cardiac tamponade, which can evolve over a relatively short period following an initially negative echocardiographic examination. Rapid bedside POCUS may facilitate early identification of a large pericardial effusion and support timely escalation of care when clinically indicated. However, as this report describes a single case, no definitive conclusions regarding diagnostic or management strategies can be drawn. Further case reports and clinical experience are needed to better characterize similar presentations and to determine the most appropriate approach to their rapid recognition and management in emergency care settings.
